# 1-(4-{2-[(*E*)-3-(4-Chloro­phen­yl)-3-oxo­prop-1-en-1-yl]phen­oxy}but­yl)-1*H*-indole-3-carbaldehyde

**DOI:** 10.1107/S1600536813002456

**Published:** 2013-01-31

**Authors:** S. Paramasivam, Santhanagopalan Purushothaman, P. R. Seshadri, Raghavachary Raghunathan

**Affiliations:** a Post Graduate and Research Department of Physics, Agurchand Manmull Jain College, Chennai 600 114, India; b Department of Organic Chemistry, University of Madras, Guindy Campus, Chennai 600 025, India

## Abstract

In the title compound, C_28_H_24_ClNO_3_, the dihedral angles between the central benzene ring and the indole ring system and the chlorobenzene ring are 70.81 (5) and 78.62 (5)°, respectively. The mol­ecular structure is stabilized by a weak intra­molecular C—H⋯O inter­action. In the crystal, pairs of C—H⋯O hydrogen bonds link the mol­ecules into inversion dimers with an *R*_2_^2^(14) motif.

## Related literature

For the biological activity of indole derivatives, see: Olgen & Coban (2003 ▶); Ho *et al.* (1986 ▶); Joshi & Chand (1982 ▶); Rodriguez *et al.* (1985 ▶); Okabe & Adachi (1998 ▶); Merck (1973 ▶). For N-atom hybridization, see: Beddoes *et al.* (1986 ▶). For a related structure, see: Paramasivam *et al.* (2012 ▶). For graph-set notation see: Bernstein *et al.* (1995 ▶). 
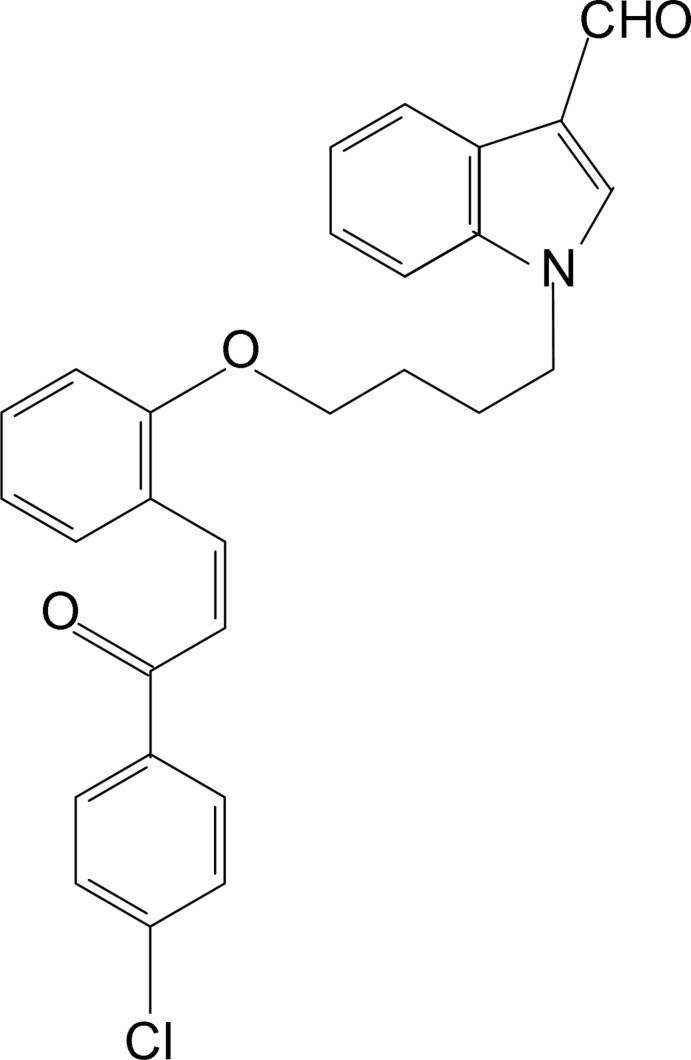


## Experimental

### 

#### Crystal data

C_28_H_24_ClNO_3_
*M* *_r_* = 457.93
Monoclinic, 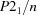

*a* = 8.7126 (3) Å
*b* = 19.1311 (6) Å
*c* = 13.9338 (4) Å
β = 93.198 (2)°
*V* = 2318.89 (13) Å^3^
*Z* = 4
Mo *K*α radiation
μ = 0.20 mm^−1^
*T* = 298 K
0.20 × 0.20 × 0.20 mm

#### Data collection

Bruker SMART APEXII area-detector diffractometer
22253 measured reflections
5782 independent reflections
4060 reflections with *I* > 2σ(*I*)
*R*_int_ = 0.026

#### Refinement

*R*[*F* ^2^ > 2σ(*F* ^2^)] = 0.049
*wR*(*F* ^2^) = 0.145
*S* = 1.03
5782 reflections
298 parameters
H-atom parameters constrained
Δρ_max_ = 0.39 e Å^−3^
Δρ_min_ = −0.46 e Å^−3^

### 

Data collection: *APEX2* (Bruker, 2008 ▶); cell refinement: *SAINT* (Bruker, 2008 ▶); data reduction: *SAINT*; program(s) used to solve structure: *SHELXS97* (Sheldrick, 2008 ▶); program(s) used to refine structure: *SHELXL97* (Sheldrick, 2008 ▶); molecular graphics: *ORTEP-3* for Windowa (Farrugia, 2012 ▶) and *PLATON* (Spek, 2009 ▶); software used to prepare material for publication: *SHELXL97*, *PLATON* and *publCIF* (Westrip, 2010 ▶).

## Supplementary Material

Crystal structure: contains datablock(s) I, global. DOI: 10.1107/S1600536813002456/kp2444sup1.cif

Structure factors: contains datablock(s) I. DOI: 10.1107/S1600536813002456/kp2444Isup2.hkl

Supplementary material file. DOI: 10.1107/S1600536813002456/kp2444Isup3.cml

Additional supplementary materials:  crystallographic information; 3D view; checkCIF report

## Figures and Tables

**Table 1:** Hydrogen-bond geometry (Å, °)

*D*—H⋯*A*	*D*—H	H⋯*A*	*D*⋯*A*	*D*—H⋯*A*
C8—H8⋯O2	0.93	2.26	2.850 (2)	121
C20—H20⋯O1^i^	0.93	2.52	3.374 (2)	152

Symmetry code: (i) 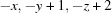.

**Fig. 1.**
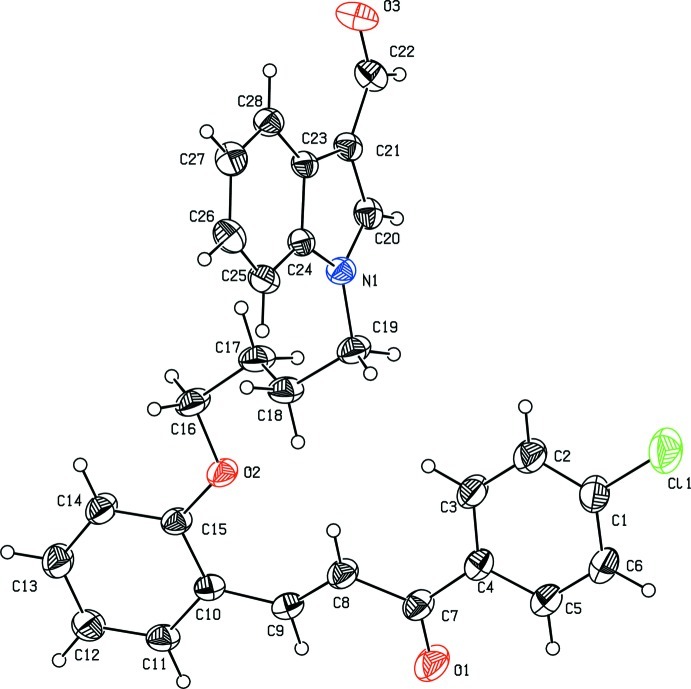
The molecular structure of the title compound, showing the atom-numbering scheme and displacement ellipsoids drawn at the 30% probability level.

**Fig. 2.**
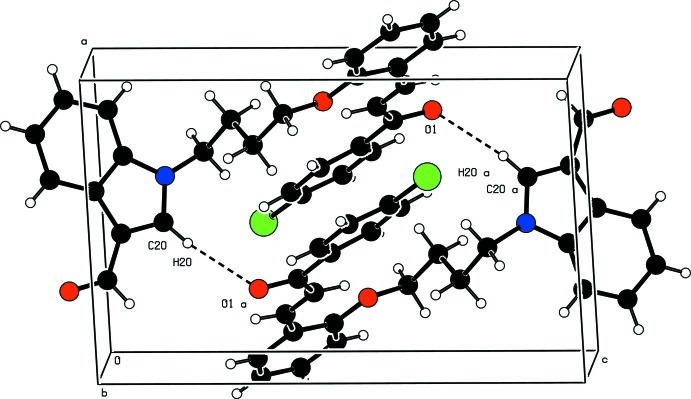
The crystal packing of the title compound. Hydrogen bonds are shown by dashed lines.

## supplementary crystallographic information

### Comment

Indole derivatives exhibit antioxidant (Olgen & Coban, 2003), central nervous system depressant and muscle relaxant properties (Ho *et al.*, 1986), antifungicidal (Joshi & Chand, 1982), antimicrobial, antiinflammatory and antiimplantation (Rodriguez *et al.*, 1985), antibacterial (Okabe & Adachi, 1998) and antihypertensive (Merck, 1973) activities. Against this background, the title compound was chosen for X-ray structure analysis (Fig. 1).

The indole ring is planar and it makes the dihedral angle with the chlorophenyl ring of 78.62 (05)°.

The sum of the bond angles around N1 [359.94 (44)°] indicates *sp*^2^ hybridization (Beddoes *et al.*, 1986). The geometric parameters of the title molecule (Fig. 1) agree well with reported similar structure (Paramasivam *et al.*, 2012).

The molecular structure is stabilised by a weak C—H···O intramolecular interaction and the crystal packing reveals a weak C—H···O hydrogen bonds (Fig. 2). In the crystal structure, the molecules at (*x*, *y*, *z*) and (- *x*, - *y* + 1, - *z* + 2) are linked by C20—H20···O1 hydrogen bond, generating a centrosymmetric dimeric ring motif *R_2_^2^*(14) (Bernstein *et al.*, 1995).

### Experimental

2 g (13.7 mmol) of 1*H*-indole-3-carbaldehyde in 25 mL dry DMF and anhydrous potassium carbonate (2 g, 13.7 mmol) were stirred for 15 min at room temperature followed by addition of 5.4 g (13.7 mmol) of (*E*)-3-(2-(4-bromobutoxy)phenyl)-1-(4-chlorophenyl)prop-2-en-1-one in 30 mL dry DMF with continued stirring for about 3 h at room temperature. After the completion of the reaction as evidenced from TLC, the solvent was filtered into crushed ice and extracted with chloroform. The organic extract was dried over Na2SO4 and concentrated under reduced pressure. Chromatography of the residue eluting with hexane/ethyl acetate mixture (8:2) gave pure (*E*)-1-(4-(2-(3-(4-chlorophenyl)-3-oxoprop-1-enyl)phenoxy)butyl)-1*H*-indole-3-carbaldehyde in good yield.

### Refinement

Hydrogen atoms were positioned geometrically and allowed to ride on their parent atoms, with C—H = 0.93 - 0.97 Å and *U*_iso_(H) = 1.5*U*_eq_(C) for methyl H atoms and 1.2 *U*_eq_(C) for other H atoms.

### Crystal data

**Table:**

C_28_H_24_ClNO_3_	*F*(000) = 960
*M**_r_* = 457.93	monoclinic
Monoclinic, *P*2_1_/*n*	*D*_x_ = 1.312 Mg m^−^^3^
Hall symbol: -P 2yn	Mo *K*α radiation, λ = 0.71073 Å
*a* = 8.7126 (3) Å	Cell parameters from 5782 reflections
*b* = 19.1311 (6) Å	θ = 1.8–28.3°
*c* = 13.9338 (4) Å	µ = 0.20 mm^−^^1^
β = 93.198 (2)°	*T* = 298 K
*V* = 2318.89 (13) Å^3^	Block, colourless
*Z* = 4	0.20 × 0.20 × 0.20 mm

### Data collection

**Table:**

Bruker SMART APEXII area-detector diffractometer	4060 reflections with *I* > 2σ(*I*)
Radiation source: fine-focus sealed tube	*R*_int_ = 0.026
Graphite monochromator	θ_max_ = 28.3°, θ_min_ = 1.8°
ω and φ scans	*h* = −8→11
22253 measured reflections	*k* = −25→21
5782 independent reflections	*l* = −18→18

### Refinement

**Table:**

Refinement on *F*^2^	Primary atom site location: structure-invariant direct methods
Least-squares matrix: full	Secondary atom site location: difference Fourier map
*R*[*F*^2^ > 2σ(*F*^2^)] = 0.049	Hydrogen site location: inferred from neighbouring sites
*wR*(*F*^2^) = 0.145	H-atom parameters constrained
*S* = 1.03	*w* = 1/[σ^2^(*F*_o_^2^) + (0.0591*P*)^2^ + 0.7822*P*] where *P* = (*F*_o_^2^ + 2*F*_c_^2^)/3
5782 reflections	(Δ/σ)_max_ < 0.001
298 parameters	Δρ_max_ = 0.39 e Å^−^^3^
0 restraints	Δρ_min_ = −0.46 e Å^−^^3^

### Special details

**Table:**

Geometry. All e.s.d.'s (except the e.s.d. in the dihedral angle between two l.s. planes) are estimated using the full covariance matrix. The cell e.s.d.'s are taken into account individually in the estimation of e.s.d.'s in distances, angles and torsion angles; correlations between e.s.d.'s in cell parameters are only used when they are defined by crystal symmetry. An approximate (isotropic) treatment of cell e.s.d.'s is used for estimating e.s.d.'s involving l.s. planes.
Refinement. Refinement of *F*^2^ against ALL reflections. The weighted *R*-factor *wR* and goodness of fit *S* are based on *F*^2^, conventional *R*-factors *R* are based on *F*, with *F* set to zero for negative *F*^2^. The threshold expression of *F*^2^ > σ(*F*^2^) is used only for calculating *R*-factors(gt) *etc*. and is not relevant to the choice of reflections for refinement. *R*-factors based on *F*^2^ are statistically about twice as large as those based on *F*, and *R*- factors based on ALL data will be even larger.

### Fractional atomic coordinates and isotropic or equivalent isotropic displacement parameters (Å^2^)

**Table:**

	*x*	*y*	*z*	*U*_iso_*/*U*_eq_	
C1	−0.0037 (3)	0.70610 (12)	0.90463 (15)	0.0675 (5)	
C2	0.0110 (3)	0.63646 (12)	0.88447 (16)	0.0743 (6)	
H2	−0.0351	0.6177	0.8284	0.089*	
C3	0.0951 (3)	0.59454 (11)	0.94836 (14)	0.0656 (5)	
H3	0.1071	0.5474	0.9341	0.079*	
C4	0.1623 (2)	0.62099 (10)	1.03343 (13)	0.0526 (4)	
C5	0.1383 (3)	0.69100 (11)	1.05371 (16)	0.0700 (6)	
H5	0.1781	0.7095	1.1116	0.084*	
C6	0.0566 (3)	0.73364 (12)	0.98970 (18)	0.0776 (7)	
H6	0.0423	0.7806	1.0039	0.093*	
C7	0.2526 (2)	0.57743 (10)	1.10492 (13)	0.0559 (4)	
C8	0.3047 (2)	0.50761 (10)	1.07578 (13)	0.0552 (4)	
H8	0.2842	0.4926	1.0129	0.066*	
C9	0.3803 (2)	0.46590 (9)	1.13797 (12)	0.0488 (4)	
H9	0.3943	0.4842	1.1997	0.059*	
C10	0.44511 (19)	0.39676 (9)	1.12663 (11)	0.0450 (4)	
C11	0.5351 (2)	0.36906 (10)	1.20364 (12)	0.0535 (4)	
H11	0.5511	0.3958	1.2590	0.064*	
C12	0.6008 (3)	0.30409 (11)	1.20085 (14)	0.0647 (5)	
H12	0.6605	0.2874	1.2533	0.078*	
C13	0.5775 (3)	0.26396 (11)	1.11985 (15)	0.0688 (6)	
H13	0.6221	0.2199	1.1174	0.083*	
C14	0.4884 (3)	0.28835 (10)	1.04196 (13)	0.0609 (5)	
H14	0.4723	0.2604	0.9878	0.073*	
C15	0.4230 (2)	0.35419 (9)	1.04394 (11)	0.0472 (4)	
C16	0.2994 (3)	0.33837 (10)	0.88721 (13)	0.0632 (5)	
H16A	0.2491	0.2958	0.9063	0.076*	
H16B	0.3921	0.3260	0.8558	0.076*	
C17	0.1931 (3)	0.38074 (11)	0.82024 (13)	0.0612 (5)	
H17A	0.1520	0.3506	0.7690	0.073*	
H17B	0.1074	0.3973	0.8556	0.073*	
C18	0.2704 (2)	0.44254 (11)	0.77648 (12)	0.0571 (5)	
H18A	0.3509	0.4258	0.7370	0.068*	
H18B	0.3183	0.4708	0.8276	0.068*	
C19	0.1603 (2)	0.48804 (10)	0.71534 (12)	0.0583 (5)	
H19A	0.0824	0.5064	0.7556	0.070*	
H19B	0.2167	0.5274	0.6911	0.070*	
C20	−0.0653 (2)	0.43252 (9)	0.62598 (12)	0.0485 (4)	
H20	−0.1354	0.4400	0.6729	0.058*	
C21	−0.09949 (19)	0.40123 (9)	0.53853 (11)	0.0445 (4)	
C22	−0.2501 (2)	0.37870 (11)	0.50518 (14)	0.0564 (5)	
H22	−0.3284	0.3832	0.5475	0.068*	
C23	0.04045 (18)	0.39990 (8)	0.48909 (11)	0.0404 (3)	
C24	0.15337 (18)	0.43141 (8)	0.55073 (11)	0.0420 (3)	
C25	0.3043 (2)	0.43976 (10)	0.52522 (13)	0.0526 (4)	
H25	0.3779	0.4609	0.5665	0.063*	
C26	0.3401 (2)	0.41551 (11)	0.43653 (14)	0.0596 (5)	
H26	0.4400	0.4207	0.4172	0.071*	
C27	0.2308 (2)	0.38327 (11)	0.37477 (13)	0.0583 (5)	
H27	0.2594	0.3668	0.3155	0.070*	
C28	0.0807 (2)	0.37520 (10)	0.39963 (11)	0.0492 (4)	
H28	0.0082	0.3538	0.3578	0.059*	
N1	0.08465 (17)	0.45088 (7)	0.63409 (9)	0.0469 (3)	
O1	0.2827 (2)	0.59973 (9)	1.18603 (10)	0.0837 (5)	
O2	0.33578 (16)	0.38163 (6)	0.96938 (8)	0.0570 (3)	
O3	−0.28421 (16)	0.35426 (9)	0.42671 (11)	0.0757 (4)	
Cl1	−0.10168 (10)	0.76018 (4)	0.82139 (5)	0.1058 (3)	

### Atomic displacement parameters (Å^2^)

**Table:**

	*U*^11^	*U*^22^	*U*^33^	*U*^12^	*U*^13^	*U*^23^
C1	0.0800 (14)	0.0584 (12)	0.0639 (12)	0.0172 (11)	0.0030 (10)	−0.0019 (10)
C2	0.0966 (17)	0.0648 (14)	0.0599 (12)	0.0149 (12)	−0.0089 (11)	−0.0127 (10)
C3	0.0897 (15)	0.0488 (11)	0.0576 (11)	0.0095 (10)	−0.0022 (10)	−0.0117 (9)
C4	0.0585 (10)	0.0466 (10)	0.0532 (9)	0.0020 (8)	0.0097 (8)	−0.0081 (8)
C5	0.0835 (15)	0.0548 (12)	0.0704 (13)	0.0083 (11)	−0.0079 (11)	−0.0187 (10)
C6	0.0954 (17)	0.0479 (12)	0.0880 (16)	0.0151 (11)	−0.0086 (13)	−0.0162 (11)
C7	0.0657 (11)	0.0528 (11)	0.0494 (9)	0.0014 (9)	0.0058 (8)	−0.0109 (8)
C8	0.0714 (12)	0.0496 (11)	0.0441 (9)	0.0032 (9)	−0.0005 (8)	−0.0074 (8)
C9	0.0542 (10)	0.0505 (10)	0.0416 (8)	−0.0067 (8)	0.0022 (7)	−0.0085 (7)
C10	0.0502 (9)	0.0462 (9)	0.0384 (7)	−0.0065 (7)	0.0006 (6)	−0.0016 (7)
C11	0.0612 (11)	0.0578 (11)	0.0405 (8)	−0.0055 (9)	−0.0066 (7)	−0.0034 (8)
C12	0.0793 (14)	0.0593 (12)	0.0532 (10)	0.0047 (10)	−0.0170 (9)	0.0061 (9)
C13	0.0935 (15)	0.0495 (12)	0.0615 (11)	0.0115 (11)	−0.0135 (11)	0.0028 (9)
C14	0.0893 (14)	0.0453 (10)	0.0466 (9)	0.0052 (10)	−0.0090 (9)	−0.0045 (8)
C15	0.0603 (10)	0.0439 (9)	0.0369 (7)	−0.0039 (8)	−0.0040 (7)	0.0006 (7)
C16	0.0968 (15)	0.0458 (10)	0.0446 (9)	−0.0043 (10)	−0.0178 (9)	−0.0066 (8)
C17	0.0779 (13)	0.0559 (12)	0.0475 (9)	−0.0123 (10)	−0.0173 (9)	−0.0022 (8)
C18	0.0674 (11)	0.0611 (12)	0.0414 (8)	−0.0094 (9)	−0.0101 (8)	−0.0029 (8)
C19	0.0799 (13)	0.0482 (11)	0.0452 (9)	−0.0041 (9)	−0.0111 (9)	−0.0047 (8)
C20	0.0539 (10)	0.0498 (10)	0.0417 (8)	0.0056 (8)	0.0022 (7)	0.0074 (7)
C21	0.0471 (9)	0.0440 (9)	0.0415 (8)	0.0005 (7)	−0.0035 (6)	0.0086 (7)
C22	0.0489 (10)	0.0632 (12)	0.0565 (10)	−0.0022 (8)	−0.0014 (8)	0.0116 (9)
C23	0.0458 (8)	0.0368 (8)	0.0377 (7)	0.0003 (6)	−0.0057 (6)	0.0084 (6)
C24	0.0482 (9)	0.0373 (8)	0.0397 (7)	0.0006 (7)	−0.0046 (6)	0.0060 (6)
C25	0.0482 (9)	0.0525 (11)	0.0560 (10)	−0.0046 (8)	−0.0082 (7)	0.0065 (8)
C26	0.0476 (10)	0.0700 (13)	0.0613 (11)	0.0019 (9)	0.0047 (8)	0.0082 (10)
C27	0.0614 (11)	0.0666 (13)	0.0473 (9)	0.0066 (9)	0.0075 (8)	0.0010 (8)
C28	0.0569 (10)	0.0498 (10)	0.0401 (8)	0.0007 (8)	−0.0051 (7)	0.0022 (7)
N1	0.0575 (8)	0.0443 (8)	0.0380 (7)	0.0004 (6)	−0.0065 (6)	0.0009 (6)
O1	0.1221 (14)	0.0712 (10)	0.0562 (8)	0.0217 (9)	−0.0091 (8)	−0.0221 (7)
O2	0.0842 (9)	0.0449 (7)	0.0396 (6)	0.0066 (6)	−0.0160 (6)	−0.0055 (5)
O3	0.0594 (8)	0.1004 (12)	0.0651 (9)	−0.0151 (8)	−0.0157 (7)	−0.0039 (8)
Cl1	0.1481 (7)	0.0834 (5)	0.0832 (4)	0.0471 (5)	−0.0170 (4)	−0.0014 (3)

### Geometric parameters (Å, º)

**Table:**

C1—C2	1.369 (3)	C16—C17	1.513 (3)
C1—C6	1.374 (3)	C16—H16A	0.9700
C1—Cl1	1.742 (2)	C16—H16B	0.9700
C2—C3	1.378 (3)	C17—C18	1.506 (3)
C2—H2	0.9300	C17—H17A	0.9700
C3—C4	1.388 (3)	C17—H17B	0.9700
C3—H3	0.9300	C18—C19	1.520 (3)
C4—C5	1.387 (3)	C18—H18A	0.9700
C4—C7	1.489 (3)	C18—H18B	0.9700
C5—C6	1.377 (3)	C19—N1	1.462 (2)
C5—H5	0.9300	C19—H19A	0.9700
C6—H6	0.9300	C19—H19B	0.9700
C7—O1	1.223 (2)	C20—N1	1.352 (2)
C7—C8	1.475 (3)	C20—C21	1.375 (2)
C8—C9	1.326 (3)	C20—H20	0.9300
C8—H8	0.9300	C21—C23	1.434 (2)
C9—C10	1.450 (3)	C21—C22	1.434 (2)
C9—H9	0.9300	C22—O3	1.211 (2)
C10—C11	1.398 (2)	C22—H22	0.9300
C10—C15	1.415 (2)	C23—C28	1.396 (2)
C11—C12	1.370 (3)	C23—C24	1.405 (2)
C11—H11	0.9300	C24—N1	1.387 (2)
C12—C13	1.371 (3)	C24—C25	1.390 (2)
C12—H12	0.9300	C25—C26	1.372 (3)
C13—C14	1.380 (3)	C25—H25	0.9300
C13—H13	0.9300	C26—C27	1.392 (3)
C14—C15	1.383 (3)	C26—H26	0.9300
C14—H14	0.9300	C27—C28	1.380 (3)
C15—O2	1.3583 (19)	C27—H27	0.9300
C16—O2	1.434 (2)	C28—H28	0.9300
			
C2—C1—C6	121.0 (2)	H16A—C16—H16B	108.6
C2—C1—Cl1	119.29 (18)	C18—C17—C16	113.45 (17)
C6—C1—Cl1	119.75 (17)	C18—C17—H17A	108.9
C1—C2—C3	119.1 (2)	C16—C17—H17A	108.9
C1—C2—H2	120.5	C18—C17—H17B	108.9
C3—C2—H2	120.5	C16—C17—H17B	108.9
C2—C3—C4	121.55 (19)	H17A—C17—H17B	107.7
C2—C3—H3	119.2	C17—C18—C19	113.20 (16)
C4—C3—H3	119.2	C17—C18—H18A	108.9
C5—C4—C3	117.70 (19)	C19—C18—H18A	108.9
C5—C4—C7	118.95 (17)	C17—C18—H18B	108.9
C3—C4—C7	123.30 (17)	C19—C18—H18B	108.9
C6—C5—C4	121.2 (2)	H18A—C18—H18B	107.8
C6—C5—H5	119.4	N1—C19—C18	113.48 (15)
C4—C5—H5	119.4	N1—C19—H19A	108.9
C1—C6—C5	119.4 (2)	C18—C19—H19A	108.9
C1—C6—H6	120.3	N1—C19—H19B	108.9
C5—C6—H6	120.3	C18—C19—H19B	108.9
O1—C7—C8	121.14 (18)	H19A—C19—H19B	107.7
O1—C7—C4	120.06 (17)	N1—C20—C21	110.35 (15)
C8—C7—C4	118.80 (15)	N1—C20—H20	124.8
C9—C8—C7	120.95 (16)	C21—C20—H20	124.8
C9—C8—H8	119.5	C20—C21—C23	106.59 (14)
C7—C8—H8	119.5	C20—C21—C22	124.54 (17)
C8—C9—C10	131.29 (16)	C23—C21—C22	128.79 (16)
C8—C9—H9	114.4	O3—C22—C21	125.64 (18)
C10—C9—H9	114.4	O3—C22—H22	117.2
C11—C10—C15	116.86 (16)	C21—C22—H22	117.2
C11—C10—C9	117.85 (15)	C28—C23—C24	119.25 (15)
C15—C10—C9	125.28 (15)	C28—C23—C21	134.26 (15)
C12—C11—C10	122.66 (17)	C24—C23—C21	106.49 (14)
C12—C11—H11	118.7	N1—C24—C25	129.92 (15)
C10—C11—H11	118.7	N1—C24—C23	107.87 (14)
C11—C12—C13	119.26 (18)	C25—C24—C23	122.20 (15)
C11—C12—H12	120.4	C26—C25—C24	117.22 (17)
C13—C12—H12	120.4	C26—C25—H25	121.4
C12—C13—C14	120.61 (19)	C24—C25—H25	121.4
C12—C13—H13	119.7	C25—C26—C27	121.62 (18)
C14—C13—H13	119.7	C25—C26—H26	119.2
C13—C14—C15	120.43 (17)	C27—C26—H26	119.2
C13—C14—H14	119.8	C28—C27—C26	121.32 (17)
C15—C14—H14	119.8	C28—C27—H27	119.3
O2—C15—C14	123.39 (15)	C26—C27—H27	119.3
O2—C15—C10	116.42 (15)	C27—C28—C23	118.38 (16)
C14—C15—C10	120.19 (16)	C27—C28—H28	120.8
O2—C16—C17	106.49 (16)	C23—C28—H28	120.8
O2—C16—H16A	110.4	C20—N1—C24	108.69 (14)
C17—C16—H16A	110.4	C20—N1—C19	125.67 (15)
O2—C16—H16B	110.4	C24—N1—C19	125.58 (15)
C17—C16—H16B	110.4	C15—O2—C16	118.49 (14)
			
C6—C1—C2—C3	−3.7 (4)	C17—C18—C19—N1	−60.3 (2)
Cl1—C1—C2—C3	176.83 (19)	N1—C20—C21—C23	0.29 (19)
C1—C2—C3—C4	1.4 (4)	N1—C20—C21—C22	−176.78 (16)
C2—C3—C4—C5	1.9 (3)	C20—C21—C22—O3	176.1 (2)
C2—C3—C4—C7	179.3 (2)	C23—C21—C22—O3	−0.3 (3)
C3—C4—C5—C6	−2.9 (3)	C20—C21—C23—C28	179.59 (18)
C7—C4—C5—C6	179.5 (2)	C22—C21—C23—C28	−3.5 (3)
C2—C1—C6—C5	2.7 (4)	C20—C21—C23—C24	−0.24 (18)
Cl1—C1—C6—C5	−177.9 (2)	C22—C21—C23—C24	176.67 (17)
C4—C5—C6—C1	0.7 (4)	C28—C23—C24—N1	−179.75 (14)
C5—C4—C7—O1	12.0 (3)	C21—C23—C24—N1	0.11 (17)
C3—C4—C7—O1	−165.4 (2)	C28—C23—C24—C25	0.9 (2)
C5—C4—C7—C8	−167.82 (19)	C21—C23—C24—C25	−179.28 (15)
C3—C4—C7—C8	14.8 (3)	N1—C24—C25—C26	−179.55 (17)
O1—C7—C8—C9	2.8 (3)	C23—C24—C25—C26	−0.3 (3)
C4—C7—C8—C9	−177.42 (18)	C24—C25—C26—C27	−0.6 (3)
C7—C8—C9—C10	−178.63 (18)	C25—C26—C27—C28	0.9 (3)
C8—C9—C10—C11	172.6 (2)	C26—C27—C28—C23	−0.3 (3)
C8—C9—C10—C15	−8.3 (3)	C24—C23—C28—C27	−0.5 (2)
C15—C10—C11—C12	0.3 (3)	C21—C23—C28—C27	179.67 (18)
C9—C10—C11—C12	179.50 (18)	C21—C20—N1—C24	−0.22 (19)
C10—C11—C12—C13	−0.3 (3)	C21—C20—N1—C19	177.14 (15)
C11—C12—C13—C14	−0.3 (4)	C25—C24—N1—C20	179.39 (17)
C12—C13—C14—C15	0.9 (4)	C23—C24—N1—C20	0.06 (18)
C13—C14—C15—O2	179.22 (19)	C25—C24—N1—C19	2.0 (3)
C13—C14—C15—C10	−0.9 (3)	C23—C24—N1—C19	−177.31 (15)
C11—C10—C15—O2	−179.82 (15)	C18—C19—N1—C20	109.0 (2)
C9—C10—C15—O2	1.1 (3)	C18—C19—N1—C24	−74.1 (2)
C11—C10—C15—C14	0.3 (3)	C14—C15—O2—C16	5.0 (3)
C9—C10—C15—C14	−178.79 (18)	C10—C15—O2—C16	−174.84 (17)
O2—C16—C17—C18	68.2 (2)	C17—C16—O2—C15	176.03 (16)
C16—C17—C18—C19	−175.60 (16)		

### Hydrogen-bond geometry (Å, º)

**Table:**

*D*—H···*A*	*D*—H	H···*A*	*D*···*A*	*D*—H···*A*
C8—H8···O2	0.93	2.26	2.850 (2)	121
C9—H9···O1	0.93	2.42	2.791 (2)	104
C20—H20···O1^i^	0.93	2.52	3.374 (2)	152

Symmetry code: (i) −*x*, −*y*+1, −*z*+2.
